# Socioeconomic disparities in survival of patients with non-muscle invasive urothelial carcinoma

**DOI:** 10.1007/s00345-024-05422-2

**Published:** 2025-02-12

**Authors:** Bohdan Baralo, Peter T. Daniels, Cody A. McIntire, Rajesh Thirumaran, John W. Melson, Asit K. Paul

**Affiliations:** 1 Division of Hematology, Oncology and Palliative Care, Department of Medicine, Virginia Commonwealth University, Leigh House (Builing), 1000 E. Clay St., Richmond, VA 23287 USA; 2 Virginia Commonwealth University School of Medicine, Richmond, VA USA; 3 Virginia Commonwealth University Massey Comprehensive Cancer Center, Richmond, VA USA; 4 Mercy Catholic Medical Center, Darby, PA USA

**Keywords:** Cancer risk factors, Urothelial, Urological oncology bladder cancer, Survival, SEER

## Abstract

**Purpose:**

Limited data are available on the impact of socioeconomic disparities on the survival of patients with non-muscle invasive urothelial carcinoma (NMIBC).

**Methods:**

We analyzed the Surveillance, Epidemiology, and End Results database to review the effects of sex, race, location, and socioeconomic factors on the survival of patients with NMIBC. We calculated 5-year overall survival (OS) and cancer-specific survival (CSS) using the log-rank test. The impact of socioeconomic factors on OS and CSS was analyzed using the Cox proportional hazards model adjusted for clinical characteristics. Hazard ratios (HR) and survival rates were reported with 95% confidence intervals (CI).

**Results:**

Analysis of 3831 patients showed that older age was associated with worse OS (HR 1.08 [1.08–1.09]) and CSS (HR 1.05 [1.04–1.06]). Women and men had similar OS (HR 0.91 [0.82–1.01]) and CSS (HR 1.12 [0.95–1.32]). Black patients had worse OS (HR 1.33 [1.08–1.62] and CSS [HR 1.54 [1.13–2.05]) than their White counterparts. Patients with an annual household income below $40,000 had worse outcomes compared to those with income above $70,000 for both OS (HR 1.79 [1.37–2.33]) and CSS (HR 1.924 [1.26–2.89]).

**Conclusions:**

There were no gender differences in survival outcomes of NMIBC. Older age, Black, American Indian/Alaskan Native, and patients with a household income below $40,000 appear to have worse survival. However, the area of residence did not seem to affect patient survival.

## Introduction

There were estimated to be 82,290 new cases of bladder cancer (BC) in the United States in 2023. The male-to-female ratio for incidence is 2–3:1, and BC is the fourth most frequently diagnosed malignancy among males in the United States. [1] The vast majority of patients have urothelial carcinoma, and up to 75% of the patients have non-muscle-invasive bladder cancer (NMIBC) at the time of diagnosis. [2] The 5-year overall survival (OS) can reach 90% when diagnosed and treated appropriately. [3] Progression to muscle-invasive or metastatic disease increases the complexity of medical care and cost. [4]

Patient demographic factors associated with inferior survival outcomes with bladder cancer include Black race, female gender, and low socioeconomic status. [5–8] However, prior studies reviewed data from older datasets with inconsistent data for Asian/Pacific Islanders and Hispanic patients. They did not review survival data for Native American/Alaskan Native patients. [9–11] While the female gender is associated with worse outcomes, it is unclear whether this is due to differences in tumor biology or delays in care. [8, 12] Surveillance, Epidemiology, and End Result (SEER) database analysis can assess the impact of patient- and disease-associated factors on clinical outcomes, clarifying existing disparities in cancer care.

In this study, we utilized the SEER database to analyze survival of the patients diagnosed with NMIBC in the last two decades to assess whether previously described disparities persist in a contemporary patient cohort. The primary goal was to review whether socioeconomic factors, including sex, race, income level, and area of residence, when adjusted for the risk of the disease, would continue to affect OS and cancer-specific survival (CSS) in patients with NMIBC.

## Methods

### Data source

The SEER database is a large collection of deidentified longitudinal data on 9 million cancer cases, with over 470,000 cases added annually. It covers approximately 30% of the population [13, 14]. For the current study, we used data from the SEER database and 17 registries (submission of November 2021). We covered patients diagnosed with NIMBC from 2004 to 2015 [15, 16]. The SEER data were de-identified and did not require Institutional Review Board approval or informed consent from patients.

### Study population

The inclusion criteria in this study were patients with bladder cancer (primary site code C67.0–C67.9), which is the primary malignancy in the patient, with urothelial carcinoma on histology records (ICD-0–3 codes 8120–8124), age 20 years and older, stage Ta, Tis, or T1, and no distant metastasis or lymph node invasion. The exclusion criteria were unknown age, race, income bracket, area of residence or survival, tumor grade, and tumor size or presence of another malignancy.

### Study outcomes and covariates

Five-year OS and CSS rates were the primary outcomes of interest, and HR was adjusted to assess the impact of socioeconomic factors on survival and detect existing disparities. OS was defined as the time from diagnosis to death from any cause, and CSS as the time from diagnosis to death from cancer as the primary outcome in our study. We assessed the following variables: sex, race, tumor extent, risk group, income, and area of residence. Race and origin were defined according to the race/ethnicity recode in the SEER: White, Black, Asian/Pacific Islander, American Indian, and Hispanic. Income was analyzed in ten thousand dollar increments, starting below the $40,000 bracket and finishing with the more than $700,000 bracket. The areas of residence were urban and rural, with further subdivision of urban areas based on population and rural area based on their proximity to urban centers. The extent of the tumor in the SEER database within the defined period of 2004–2015 was recorded according to the American Joint Commission on Cancer (AJCC) 6th edition: Ta, Tis, T1.

### Risk stratification

In our statistical analysis, patients were divided patients into three risk groups: low-risk (low-grade solitary Ta less than 3 cm), intermediate-risk (solitary low-grade Ta larger than 3 cm, high-grade Ta, low-grade T1), and high-risk (high-grade T1, high-grade Ta larger than 3 cm, carcinoma in situ). This grouping was performed in a similar fashion to the study by Munseok and Langabeer to perform a more accurate calculation of risk estimates for socioeconomic factors. [9] This risk stratification differs from that proposed by the American Urology Association/Society of Urologic Oncology (AUA/SUO), as it does not include recurrence time, number of tumors on initial presentation, Bacille Calmette-Guerin (BCG) failure, variant histology, lymphovascular invasion, or prostatic urethra involvement as this information is not available in the SEER database. [17]

### Statistical analysis

Descriptive statistics were calculated for all variables using the statistical software GraphPad PRISM 10.1 (San Diego, CA, USA). We used the log-rank test to calculate the 5-year OS and CSS in the different groups and reported them with 95% confidence intervals (CI). We calculated the adjusted hazard ratios (HR) using Cox proportional hazard regression.

## Results

We found 3831 patients in the SEER database (2005–2015) who satisfied our predefined inclusion and exclusion criteria. The median age of the patients at diagnosis was 71 years (interquartile range (IQR): 62–80). Every additional year of age at the time of diagnosis was associated with an 8% increase in the overall risk of death (hazard ratio [HR], 1.08; 95% CI 1.08–1.09; p < 0.001) and a 5% increase in cancer-specific death (HR, 1.05; 95% CI 1.04–1.06; p < 0.001).

**Fig. 1:**
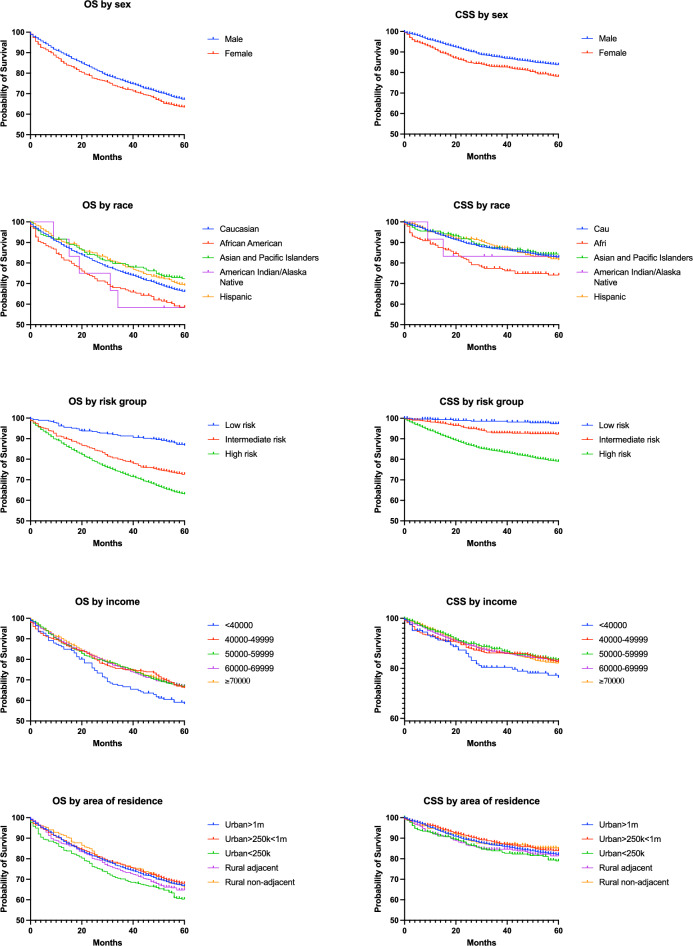
OS and CSS of patients with NMBC based on sex, race, risk group, income and area of residence

In our study, 75.57% of patients with NMIBC were men. The 5-year OS and CSS were 3.8% and 5.7% better for men (Table 2, Fig. 1). However, the adjusted HR did not show a statistically significant survival difference between the sexes (Table 3).

In our study, 82% of the patients were White. Hispanics, Asian/Pacific Islanders, and Black patients represented 5–7% of the population. The American Indian/Alaskan Native group was by far the smallest racial group included in our analysis, with only 12 patients (Table 1).

**Table 1:** Demographic, socioeconomic and clinical characteristics of NMIBC patients

Variable	Patient number, % (n = 3831)
Age (median (IQR)	71 (62–80)
Sex	
Male	75.57% (2895)
Female	24.43% (936)
Race/ethnicity	
White	81.94% (3139)
Black	4.99% (191)
America Indian/Alaskan Native	0.31% (12)
Asian/Pacific Islanders	6% (230)
Hispanics	6.76% (259)
Stage	
Ta	15.11% (579)
Tis	17.1% (655)
T1	67.79% (2597)
Grade	
Well differentiated (Grade 1)	10.21% (391)
Moderately differentiated (Grade 2)	19.86% (761)
Poorly differentiated (Grade 3)	24.04% (921)
Undifferentiated (Grade 4)	45.89% (17.58%)
Risk group	
Low risk	7.6% (291)
Intermediate risk	15.01% (575)
High risk	77.39% (2965)
Size of the tumor	
< = 3	60.27% (2309)
> 3	39.37% (1522)
Household income	
< 40,000	4.36% (167)
40,000–49,999	10.55% (404)
50,000–59,999	17.52% (671)
60,000–69,999	29.44% (1128)
> 70,000	38.14% (1461)
Population of the residential area	
Urban > 1,000,000	52.7% (2019)
Urban 250,000–1,000,000	24.25% (929)
Urban < 250,000	8.22% (315)
Rural adjacent to urban area	8.2% (314)
Rural not adjacent to urban	6.63% (254)

Among the patients in our study, 77.4% were in the high-risk group, 15% in the intermediate group, and 7.6% in the low-risk group. Patients with low-risk diseases had the best outcomes (Table 3). The calculated adjusted HR had the strongest impact on survival compared to socioeconomic risk factors for both OS and CSS. (Table 2).

**Table 2:** 5-year survival of NMIBC patients based on race, income, area of residence and risk group

	Overall survival	Cancer specific survival
	5-year survival rate (%), [95% CI]	5-year survival rate (%), [95% CI]
Sex		
Male	67.17 [65.47;68.93]	83.77 [82.4;85.25]
Female	63.34 [60.31;66.56]	78.018 [75.36;80.982]
Race/ethnicity		
White	66.04 [64.4;67.84]	82.77 [81.42;84.23]
Black	58.52 [51.74;65.99]	74.16 [68.27;81.41]
Hispanic	69.13 [63.74;75.34]	81.54 [77.02;87.27]
Asian/Pacific Islander	72.44 [67.07;78.79]	84.38 [80.11;90.04]
American Indian/Pacific Islander	58.33 [36.57;89.65]	69.44 [49.27;100]
Risk group		
Low risk	86.79 [83.32;91.36]	97.34 [95.956;100]
Intermediate risk	72.66 [69.18;76.54]	92.22 [90.2;94.91]
High risk	62.99 [61.26;64.78]	78.96 [77.44;80.57]
Income		
< $ 40,000	58.41 [61.27;66.39]	76.323 [70.2;84.15]
$40,000-$49,999	66.12 [61.7;71.01]	82.75 [79.25;87.03]
$50,000-$59,000	66.65 [63.2;70.39]	83.32 [80.55;86.58]
$60,000-$69,000	66.85 [64.15;69.72]	82.88 [80.68;85.36]
> 70,000	66.52 [64.13;69.03]	82.1 [80.13;84.3]
Area of residence (by population)		
Urban > 1,000,000	66.66 [64.63;68.8]	82.1 [80.4;83.95]
Urban 250,000–1,000,000	67.94 [65.01;71.08]	83.8 [81.45;86.5]
Urban < 250,000	60.33 [55.14;65.94]	78.92 [74.55;84.24]
Rural adjacent to urban area	64.77 [59.72;70.38]	81.36 [77.29;86.4]
Rural not adjacent to urban	65.89 [60.36;72.16]	84.72 [80.69;90.02]

The lowest 5-year OS was noted in Black and American Indian/Alaskan Native patients, at 7.48% and 7.7% lower than that in White patients, respectively (Table 2, Fig. 1). The 5-year CSS rate was 8.6% lower in Black patients compared to White patients. Multivariate analysis indicated increased HR for OS and CSS for both Black and American Indian/Alaskan Native patients (Table 3), and no difference in OS and CSS was noted for Hispanic and Asian/Pacific Islander patients.

**Table 3:** Cox regression analysis of overall and cancer-specific survival in patients with NMIBC

Variable	Overall survival		Cancer specific survival	
	HR (95% CI)	p-value	HR (95% CI)	p-value
Age	**1.08 [1.08–1.09]**	** < 0.001**	**1.05 [1.04–1.06]**	** < 0.001**
Sex				
Male	Reference		Reference	
Female	0.91 [0.82–1.01]	0.09	1.12 [0.95–1.32]	0.18
Race				
White	Reference		Reference	
Black	**1.33 [1.08–1.62]**	**0.007**	**1.54 [1.13–2.05]**	**0.004**
Hispanic	0.89 [0.72–1.08]	0.24	1.07 [0.79–1.42]	0.65
Asian/Pacific Islander	0.82 [0.66–1.01]	0.07	0.98 [0.69–1.33]	0.89
American Indian/Alaskan Native	**4.11 [1.87–7.71]**	** < 0.001**	**3.36 [1.04–7.92]**	**0.02**
Risk group				
Low risk	Reference		Reference	
Intermediate risk	**1.88 [1.41–2.42]**	** < 0.001**	**3.84 [1.95–8.71]**	** < 0.001**
High risk	**2.1 [1.66–2.71]**	** < 0.001**	**8.27 [4.42–18.8]**	** < 0.001**
Income				
> 70,000	Reference		Reference	
< $ 40,000	**1.79 [1.37–2.33]**	** < 0.001**	**1.92 [1.26–2.89]**	**0.002**
$40,000-$49,999	1.2 [0.99–1.44]	0.06	1.21 [0.9–1.62]	0.2
$50,000-$59,000	1.11 [0.96–1.29]	0.16	1.01 [0.79–1.27]	0.97
$60,000-$69,000	1.05 [0.94–1.18]	0.4	1 [0.84–1.2]	0.99
Area of residence (by population)				
Urban > 1,000,000	Reference		Reference	
Urban 250,000–1,000,000	1.01 [0.89–1.13]	0.94	0.89 [0.73–1.07]	021
Urban < 250,000	1.01 [0.84–1.2]	0.92	1.11 [0.83–1.46]	0.48
Rural adjacent to urban area	0.94 [0.77–1.14]	0.53	0.92 [0.67–1.26]	0.61
Rural not adjacent to urban	0.93 [0.74–1.17]	0.56	0.7 [0.47–1.04]	0.08

Statistically significant findings are highlighted in bold

Household income was skewed toward higher incomes and areas with a higher population (Table 1). Patients with household incomes below $40,000 had 5-year OS 7.7–8.43% lower and 5-year CSS 5.7–7% lower than patients whose household income was above the specified threshold (Table 2, Fig. 1). Multivariate regression confirmed that only patients with a household income below $40,000 had an increased risk of premature death (Table 3).

While the 5-year OS varied from 60.326 to 67.938% and 5-year CSS varied from 78.917 to 84.721 (Table 2), multivariate analysis did not reveal any survival benefit in patients from urban versus rural areas (Table 3).

## Discussion

This SEER database analysis assessed the association between patient characteristics and survival outcomes in a contemporary NMIBC cohort. The disease risk category was strongly correlated with survival outcomes. Significant associations were observed for patient age, race, household income, and disease risk category. Patient sex and residential area were not independently associated with survival outcomes.

Our observed association between age, black race, and household income is consistent with studies of earlier cohorts. The results of our analysis are consistent with those of the studies performed by Seo et al. and Sung et al., which showed advanced age as a predictive factor of worse survival in patients with NMIBC. [9, 10] Additionally, prior studies reported that Black patients are more likely to present with higher stages and grades of bladder cancer than White patients, leading to worse survival. However, data regarding survival after adjusting for race and other socioeconomic factors remain controversial, with some studies reporting that survival disparities remain, whereas others report comparable CSS. [4–6, 18, 21–24] In our study, we confirmed that Black patients have worse OS and CSS compared to White patients.

A review of the California registry by Lara et al. and the SEER database analysis performed by Sung et al. showed that patients with low socioeconomic status had worse OS and CSS. [10, 23] This corresponded to our findings of worse OS and CSS in patients with household incomes below $40,000. These findings further emphasize the need to understand better factors contributing to worse outcomes in patients with NMIBC. The mechanism by which low socioeconomic status affects cancer outcomes is debated in the literature. Higher incidence of nicotine dependence that is strongly associated with urothelial cancer, a higher burden of other comorbidities likely to limit survival, delayed presentations, and more advanced disease at the time of diagnosis, the impact of financial toxicity on treatment options, and worse surgical outcomes are among the factors considered to be responsible for this disparity. [26, 27] Given the inconsistency in the published literature, the exact mechanism driving disparities is still under investigation.

Women are known to present with a higher grade and stage of BC at the time of diagnosis, have more aggressive tumor behavior, and experience delays in work-up. These factors likely contribute to the worse outcomes in women. [5, 6, 8, 11, 12, 18–20] Even among patients with NMIBC, recent SEER databases reviewed by Seo et al., which cover 1988–2006 data, showed better OS in men (HR, 0.96; 95% CI: 0.93–0.99). [9] This represents the small but existing difference in outcomes between men and women with early-stage disease. In our study, we observed minimal differences in 5-year OS and CSS between men and women, which did not reveal a statistically significant difference after multivariate analysis. (Table 2, 3). This could be linked to awareness of such disparities among men and women for over two decades. Our database review covers patients diagnosed and treated after 2004, which might reflect changing practices in early screenings and diagnosis of women who present to medical practitioners with lower primary tract symptoms.

Data on the survival of Hispanic patients with NMIBC in the literature are inconsistent. For example, in the SEER database analysis performed by Seo et al., OS was lower compared to White patients. However, in a review of the California Cancer Registry by Sung et al. Hispanic patients were found to have better OS (HR, 0.93; p 0. 0047) and CSS (HR, 0.92; p < 0.001), and the SEER database review by Yee et al. did not find any difference in OS (HR, 1.03; 95% CI:0.97–1.1) for Hispanic patients. [9, 10, 18] The results of our study support the data from a later study by Yee et al. and do not show any statistically significant difference in OS and CSS in Hispanics.

In our study, the 5-year OS and CSS of Asians and Pacific Islanders with NMIBC resembled those of Hispanics, with a slight improvement in OS but not CSS. No significant difference was noted in our study, which is consistent with the results of the SEER database review performed by Yee et al. (OS (HR, 0.95; 95% CI: 0.89–1.02). [18]

It is known that bladder cancer is rare in American Indian and Alaskan natives, but mortality is higher if they are diagnosed. [21] In our study, we noticed that the HR for both OS and CSS for American Indians and Alaskan Natives was highest among all racial groups in the SEER database. However, only 12 patients were included in our analysis, which is low number to draw a definite conclusion about this association. Unfortunately, no large study on NMIBC outcomes in Native Americans is available in the literature. Further investigation is needed to confirm our findings and define the factors leading to worse survival in these populations.

Adequate access to care is one factor that can improve the outcomes of cancer patients. Cancer mortality rates are higher in rural versus urban areas in the United States. This difference is especially noticeable in cancers for which effective early detection methods and treatment options are available. [22] The applicability of the above method for NMIBC was previously tested in studies performed by Deuker et al. and Lara et al. These studies did not show any difference in mortality between rural and urban populations. [23, 24] The results of our study were consistent with those previously reported. This reassures that most patients can continue to follow up with local cancer centers after being diagnosed with NMICB, which should improve access and compliance and decrease the financial burden.

Several study limitations should be noted. Retrospective database analysis is at risk of selection bias. Selection bias is present due to the retrospective nature of the database analysis. There is also a possibility of miscoding during data entry or histological misclassification. If a patient had a procedure or treatment outside of the location that participated in the SEER data collection, this information may be missing as well [13, 25, 26] The clinical risk stratification scheme used in this study does not strictly follow AUA guidelines as certain AUA risk stratification criteria (BCG treatment history, number of tumors at initial presentation, and number of recurrences) are not available in the SEER database. Even though our studies showed that a higher-risk group is associated with worse OS and CSS, it should be interpreted with caution (Table 3), as improved stratification can magnify differences in the OS and CSS between groups and thus affect study results.

The current study confirms ongoing disparity in outcomes for early-stage bladder cancer among Black patients and patients with low household income, which warrants healthcare policies supporting equitable healthcare resources and targeted interventions such as outreach programs for early diagnosis and intervention. Representation of Black patients in clinical trials was historically low, and while we see some improvement over time, further effort is needed to ensure adequate representation in clinical trials. [27]

## Conclusion

Our review of the latest SEER database showed no gender difference in survival outcomes of NMIBC. Patient characteristics associated with worse survival are older age, Black race, and household income < $40,000. The size of the patient’s residential area does not affect the survival of patients with NMIBC. The number of Native American/Alaskan Native patients included in our study was very low. However, the trend for poor overall and cancer-specific survival in this group is concerning and warrants additional studies to verify this finding.

## Data Availability

The datasets generated during the current study are available in the Surveillance, Epidemiology, and End Results Program (https://seer.cancer.gov).
